# Attenuated Inflammatory Response in Aged Mice Brains following Stroke

**DOI:** 10.1371/journal.pone.0026288

**Published:** 2011-10-18

**Authors:** Matthias W. Sieber, Ralf A. Claus, Otto W. Witte, Christiane Frahm

**Affiliations:** 1 Hans Berger Department of Neurology, Jena University Hospital, Jena, Thuringia, Germany; 2 Centre for Sepsis Control and Care, Jena University Hospital, Jena, Thuringia, Germany; Julius-Maximilians-Universität Würzburg, Germany

## Abstract

**Background:**

Increased age is a major risk factor for stroke incidence, post-ischemic mortality, and severe and long-term disability. Stroke outcome is considerably influenced by post-ischemic mechanisms. We hypothesized that the inflammatory response following an ischemic injury is altered in aged organisms.

**Methods and Results:**

To that end, we analyzed the expression pattern of pro-inflammatory cytokines (TNF, IL-1α, IL-1β, IL-6), anti-inflammatory cytokines (IL-10, TGFβ1), and chemokines (Mip-1α, MCP-1, RANTES) of adult (2 months) and aged (24 months) mice brains at different reperfusion times (6 h, 12 h, 24 h, 2 d, 7 d) following transient occlusion of the middle cerebral artery. The infarct size was assessed to monitor possible consequences of an altered inflammatory response in aged mice. Our data revealed an increased neuro-inflammation with age. Above all, we found profound age-related alterations in the reaction to stroke. The response of pro-inflammatory cytokines (TNF, and IL-1β) and the level of chemokines (Mip-1α, and MCP-1) were strongly diminished in the aged post-ischemic brain tissue. IL-6 showed the strongest age-dependent decrease in its post-ischemic expression profile. Anti-inflammatory cytokines (TGFβ1, and IL-10) revealed no significant age dependency after ischemia. Aged mice brains tend to develop smaller infarcts.

**Conclusion:**

The attenuated inflammatory response to stroke in aged animals may contribute to their smaller infarcts. The results presented here highlight the importance of using aged animals to investigate age-associated diseases like stroke, and should be considered as a major prerequisite in the development of age-adjusted therapeutic interventions.

## Introduction

In general, the interplay between the peripheral immune system and the brain is well balanced in young organisms but becomes unfavorable with ageing [1]. Impaired immune homeostasis in aged organisms is associated with a decline in the ability to adapt to environmental stress. The consequence of this is a significant higher vulnerability for diseases and their impacts in the elderly. Stroke is the most common and most important vascular disease of the cerebral nervous system. Cerebral infarcts are the second leading cause of death worldwide and the leading cause of adult disability. Increased age is a major risk factor for stroke incidence, post-ischemic mortality, and severe and long-term disability [2], [3], [4]. Although stroke is an extremely important health issue, most pharmaceutical companies have terminated their research programs due to the failure of previous studies [5]. One explanation for the inability to transfer experimental results to the clinic may be the predominant incorporation of young rodents in basic research. Several initial indications that the response of the central nervous system to an ischemic event is age dependent have been reported [6], [7], [8], [9]. However, post-ischemic mechanisms are complex and more fundamental studies are required to understand the impact of age on these processes.

Following cerebral ischemia, a cascade of inflammatory mediators including cytokines (TNF, IL-1α, IL-1β, IL-6, IL-10, TGFβ1) and chemokines (MCP-1 [CCL2], Mip-1α [CCL3], RANTES [CCL5]) is initiated. Cytokines and chemokines have complex, overlapping and pleiotropic functions that may be both beneficial and deleterious. The temporal expression profile of each inflammatory mediator, its specific cell source and target, and their coordinated interaction are important for stroke recovery. The complex as well as dual role (beneficial versus deleterious) of the inflammatory response to stroke has been extensively discussed [10], [11], [12], [13]. Ageing is associated with alterations of the cerebral inflammatory system [14], [15], [16], [17]. In particular, TNF, IL-1, and IL-6 have been found to be increased with age [7], [18]. The inflammatory reaction of aged brains to cerebral injuries has received little attention to date [19], [20], [21], [22]. Indeed, the response to stroke is currently unknown. It is assumed that the inflammatory reaction influences the dimension of the injury [20]. Previous studies investigating the infarct size in old mice have revealed conflicting results; there are reports of increased, decreased or similar infarct sizes in aged brains [8].

We hypothesized that the extent and the progression of the immune response following an ischemic injury are altered in the elderly and that this age-dependent inflammatory reaction is a determinant of the outcome following stroke. The aim of the present study was to systematically characterize the age-dependent changes of the infarct size and the inflammatory response in an experimental stroke model of mice. Transient occlusion of the middle cerebral artery (MCAO) – a model that closely resembles human stroke [23] – was used to induce cerebral infarction. The infarct size was analyzed at three different reperfusion times up to 7 days. The expression patterns of pro-inflammatory cytokines (TNF, IL-1α, IL-1β, IL-6), anti-inflammatory cytokines (IL-10, TGFβ1), and chemokines (Mip-1α, MCP-1, RANTES) were investigated at different reperfusion times (6 h, 12 h, 24 h, 2 d, 7 d).

## Materials and Methods

All investigated mice were taken from a C57/BL6 inbred mice strain, originally obtained from Jackson Laboratory, and randomly assigned to the groups (described in more detail in Supplement S1). Adult (2 months), middle-aged (9 months), and aged (15 months and 24 months) male native mice were used to investigate age-related neuro-inflammation (n = 4, each). The age-dependent inflammatory response following induction of a cerebral infarct (6 h, 12 h, 24 h, 2 d, and 7 d) was studied using adult (2 months) and aged (24 months) mice that had undergone MCAO (n = 4, each). Infarct volumes were analyzed 2 h, 2 d, and 7 d after MCAO (n = 4, each). MCAO was performed as described previously [24]. Briefly, a monofilament was introduced into the internal carotid artery through an incision of the left common carotid artery. In this position, the middle cerebral artery was occluded for 30 min. Up to 7 d after reperfusion, the mortality rates were 7% and 39% in adult and aged mice, respectively. The effect of the surgical procedure was controlled using sham-operated mice (n = 4, each). These animals underwent anesthesia and surgical procedures similar to the MCAO group but without occlusion of the middle cerebral artery. In a pre-study, the cerebral blood flow (CBF) was measured by laser Doppler flowmetry (LDF) (Peri Flux System 5000, Perimed, Sweden) in adult (n = 10) and aged (n = 9) mice. Rectal temperature was measured using the DC Temperature Control System (FHC Inc., USA) in adult (n = 6) and aged (n = 8) mice. Animals were maintained with access to water and food *ad libitum*. All animal procedures were approved by the local government (Thueringer Landesamt für Lebensmittelsicherheit und Verbraucherschutz [TLLV], Dep.2, Gesundheitlicher Verbraucherschutz, Veterinärwesen, Pharmazie, Germany, Approval ID's 02-20/05 and 02-028/10) and conformed to international guidelines on the ethical use of animals. All surgeries were performed under deep anesthesia (isoflurane).

### Sample preparation

Infarct volumes were analyzed on Map2 immunostained slices [2 h, 2 d, and 7 d after reperfusion, as described in detail by us previously [25]]. Deeply anesthetized mice were fixed by perfusion through the ascending aorta with 4% paraformaldehyde. Brains were removed, cryoprotected in 0.2 M phosphate-buffered saline containing 30% sucrose, and stored at −80°C. Coronal sections (40-µm thick) were cut with a freezing microtome (MH400, Microm International GmbH).

For mRNA and protein quantification, a separate group of mice underwent MCAO. Animals were decapitated under deep anesthesia and brains were removed at 6 h, 12 h, 24 h, 2 d, and 7 d. Using a Precision Brain Slicer (BS-2000C Adult Mouse, Braintree Scientific Inc.), three coronal sections were dissected cutting +2.8 and +0.8 mm to bregma (rostral slice), +0.8 and −1.2 mm to bregma (middle slice), and −1.2 and −3.2 mm to bregma (caudal slice). The ipsilateral (ischemic tissue) and contralateral (non-ischemic tissue) hemispheres of the middle brain slice were separated and snap-frozen (for details see Supplement S1). Adjacent slices were used for infarct validation (Map2 immunohistochemistry), to select mice with similar lesion size for mRNA and protein quantification.

Tissue samples of native mice (right hemisphere, +0.8 and −1.2 mm to bregma) were used to study the age-related cerebral inflammation.

For quantitative PCR (qPCR), tissue samples were homogenized in 1 ml QIAzol Lysis Reagent and total RNA was isolated using the RNeasy Lipid Tissue Mini Kit (Qiagen GmbH). For cytokine bead assay (CBA), tissue samples were homogenized in 400 µl ice-cold lysis buffer (0.32 M sucrose; 4 mM Tris-HCl, pH 7.4; 1 mM EDTA; and 0.25 mM dithiothreitol) containing protease inhibitors Complete Mini (Roche) and proteins were isolated as previously described [26].

### Immunohistochemistry (Map2)

Free-floating sections (cerebrum, 40 µm thick) were incubated at 4°C overnight with antibody against Map2 (monoclonal mouse anti-MAP2 (2a+2b), clone AP-20, Sigma-Aldrich, 1∶1,000), then further processed by the Vectastain Elite ABC Kit (Vector Laboratories) using a biotinylated secondary antibody (donkey anti-mouse, Dianova, 1∶500). Immunoreactivity was developed in 3,3′-diaminobenzidine tetrahydrochloride (DAB; Sigma).

### qPCR

For qPCR, equal amounts of total RNA (1 µg) were transcribed in cDNA with RevertAid First Strand cDNA synthesis kit (Fermentas [27]). Amplification products were analyzed using gel electrophoresis, melting curve analysis, and sequencing to confirm the PCR product specificity. PCR was performed in a volume of 20 µl containing Brilliant® II SYBR Green® qPCR Master Mix (Stratagene), cDNA (equivalent to 25 ng reverse transcribed RNA), and specific primers (Table 1) each at a final concentration of 500 nM. Amplification was performed using the Rotor-Gene 6000 (Corbett Life Science) applying the following cycle conditions: 10 min polymerase activation, 40 amplification cycles (95°C for 30 s, 60°C for 30 s, 72°C for 30 s), and melting curve.

**Table 1:** Primer sequences.

Primer	Sequence (5′→3′)	GenBank accession number
Gapdh forward	CAACAGCAACTCCCACTCTTC	NM_008084.2
Gapdh reverse	GGTCCAGGGTTTCTTACTCCTT	
Hmbs forward	GAAATCATTGCTATGTCCACCA	NM_013551.2
Hmbs reverse	GCGTTTTCTAGCTCCTTGGTAA	
TNFα forward	GTCTACTGAACTTCGGGGTGAT	NM_013693.2
TNFα reverse	ATGATCTGAGTGTGAGGGTCTG	
IL-1α forward	GCCTTATTTCGGGAGTCTAT	NM_010554.4
IL-1α reverse	TAGGGTTTGCTCTTCTCTTACA	
IL-1β forward	GAAGAGCCCATCCTCTGTGA	NM_008361.3
IL-1β reverse	TTCATCTCGGAGCCTGTAGTG	
IL-6 forward	ACAAAGCCAGAGTCCTTCAGAG	NM_031168.1
IL-6 reverse	CATTGGAAATTGGGGTAGGA	
IL-10 forward	ATGGTGTCCTTTCAATTGCTCT	NM_010548.1
IL-10 reverse	AGGATCTCCCTGGTTTCTCTTC	
TGFβ1 forward	TGCTTCAGCTCCACAGAGAA	NM_011577.1
TGFβ1 reverse	TACTGTGTGTCCAGGCTCCA	
Mip-1α forward	TGGAACTGAATGCCTGAGAGT	NM_011337.2
Mip-1α reverse	TAGGAGATGGAGCTATGCAGGT	
MCP-1 forward	AGGTGTCCCAAAGAAGCTGTAG	NM_011333.3
MCP-1 reverse	AATGTATGTCTGGACCCATTCC	
RANTES forward	CCAGAGAAGAAGTGGGTTCAAG	NM_013653.2
RANTES reverse	AAGCTGGCTAGGACTAGAGCAA	

The GenBank accession numbers were obtained from the NCBI (March 2010).

### Cytokine bead assay (CBA)

The protein expression pattern was measured by a CBA with TNF, IL-1α, IL-1β, IL-6, IL-10, Mip-1α, MCP-1, and RANTES mouse Flex Sets (BD Bioscience). No antibody was available for TGFβ1. The procedure was performed according to the manufacturer's instructions with the following modifications. To ensure a valid analysis of proteins below 10 pg/ml (the default outlined limit for quantification), (i) 300 µg whole protein was dissolved in 50 µl lysis buffer, (ii) the standard curve was constructed from 1.25–156 pg/ml (dissolved in lysis buffer), and (iii) samples as well as standards were washed twice at the end of the procedure to reduce background signals. Standards and test samples were analyzed using the Cytomics FC500 Flow Cytometry System (Beckman Coulter) with its settings optimized to ensure valid quantifications even for proteins present in very low amounts.

### Data analysis

#### qPCR

External standard curves of purified PCR products (five 10-fold dilution series) were applied for absolute quantification, as described previously [27]. The numbers of transcripts were calculated per 1,000 transcripts of *Gapdh* by including the criterion of the length of each specific amplicon. *Hmbs* was used as a second internal standard. Both *Gapdh* and *Hmbs* were stably expressed under the conditions of investigation.

#### CBA

The raw data from the Cytomics FC500 Flow Cytometry System were analyzed using FCAP Array™ software (SoftFlow). Protein concentrations of the test samples were calculated using a corresponding standard curve (supplied recombinant protein with known concentration).

### Statistical analysis

Differences between the infarct volumes of adult (2 months) and aged (24 months) mice at different time points (2 h, 2 d, and 7 d) were analyzed with ANOVA and post-hoc Tukey test. Significant differences of mRNA and protein expression values dependent on ageing *per se* (versus 2-month-old controls) were calculated with ANOVA and post-hoc Tukey test (& p≤0.05, && p≤0.01, &&& p≤0.001). Correlation of the expression level with age (R, Pearson correlation) is indicated by + p≤0.05, ++ p≤0.01, and +++ p≤0.001. Significant post-ischemic differences of the expressions levels (ipsi versus contra) at several time points (6 h, 12 h, 24 h, 2 d and 7 d) were calculated with paired two-way ANOVA and post-hoc Tukey test. Analyses were done separately for adult (* p≤0.05, ** p≤0.01, *** p≤0.001) and aged (# p≤0.05, ## p≤0.01, ### p≤0.001) mice. Significant age-dependent differences of absolute expression within the ipsilateral hemisphere were calculated within a further two-way ANOVA and are displayed by † p≤0.05, †† p≤0.01, ††† p≤0.001. All tests were performed with SigmaStat (software version 3.5).

## Results

Almost all the tested inflammatory mediators showed increased expression values during ageing *per se*. The pro-inflammatory cytokines, particularly TNF, as well as the chemokines Mip-1α and MCP-1 showed a distinct up-regulation with age. Following stroke, the expression of almost all the tested inflammatory mediators was significantly up-regulated in ischemic tissues. However, a clearly attenuated inflammatory response was apparent in aged brains following the ischemic insult. TNF, IL-1α, IL-1β, IL-6, Mip-1α, and MCP-1 displayed an age-related decreased post-ischemic expression, with IL-6 exhibiting the strongest decrease. Aged mice brains also exhibited smaller infarcts. The sham procedure had no substantial effect on the expression of all the tested cytokines and chemokines. No significant differences of the cerebral blood flow and the body temperature were observed between adult and aged mice during or after MCAO (data not shown).

### Infarct volume

**Figure 1:**
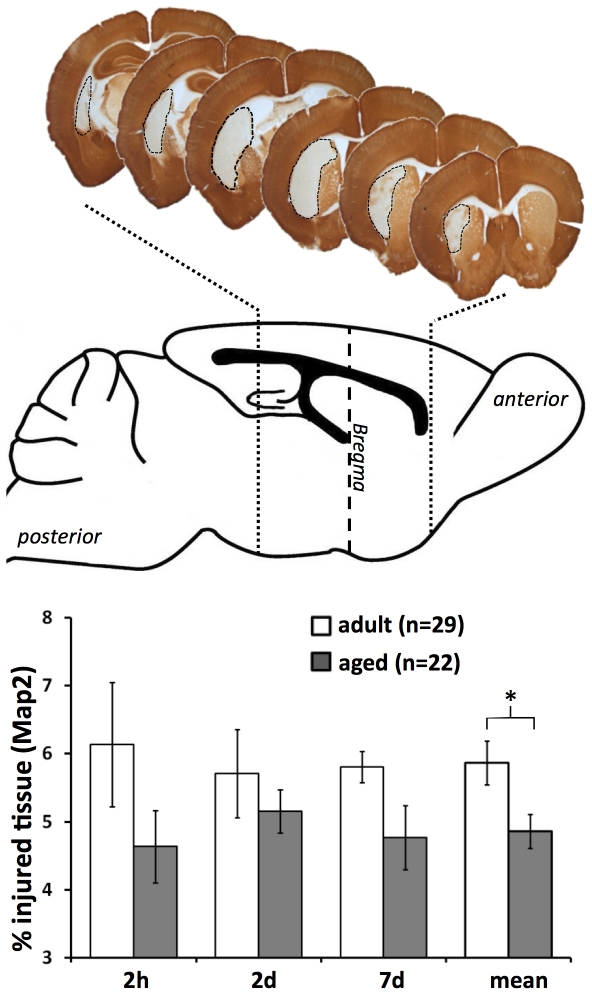
Infarct volumes at different ages. The mean infarct volume (±SEM) was significantly smaller in aged mice brains (Map2 immunohistochemistry, ANOVA with post-hoc Tukey test, * p≤0.05).

Occlusion of the middle cerebral artery for 30 min led to an ischemic injury mainly restricted to striatal regions of the mice brains. At different reperfusion times (2 h, 2 d, and 7 d) the infarct size tend to be smaller in aged mice. Taking all time points together, the mean infarct volume of aged mice was significantly smaller (Figure 1, Map2 immunohistochemistry, ANOVA with post-hoc Tukey test, p≤0.05).

### Pro-inflammatory cytokines TNF, IL-1α, and IL-1β

**Figure 2:**
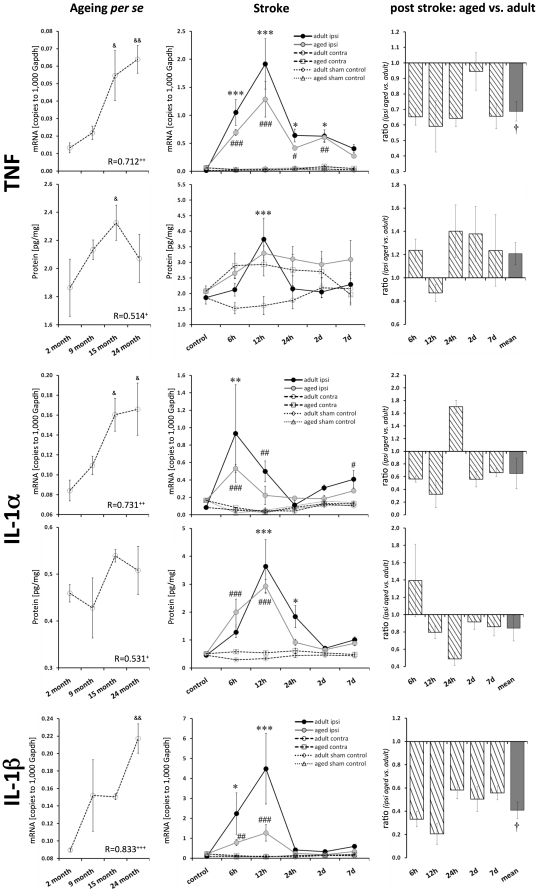
Age- and stroke-dependent expression of TNF, IL-1α, and IL-1β. TNF, IL-1α, and IL-1β displayed significantly elevated expression levels with age. All these pro-inflammatory cytokines showed a distinct response following stroke, which was attenuated in aged brains. Expression values are shown as mean ± SEM and ratios as geometric mean ± SEM. Significant age-related differences (versus 2-month-old native mice) are indicated by & p≤0.05, && p≤0.01, and &&& p≤0.001 (ANOVA and post-hoc Tukey test). Correlation of the cytokine expression level with age is indicated by + p≤0.05, ++ p≤0.01, and +++ p≤0.001 (R, Pearson correlation). Significant post-stroke differences (ipsi versus contra) are indicated by adult: * p≤0.05, ** p≤0.01, and *** p≤0.001; and aged: # p≤0.05, ## p≤0.01, and ### p≤0.001 (paired two-way ANOVA and post-hoc Tukey test). Significant age-dependent differences after stroke (ipsi aged versus ipsi adult) are displayed by † p≤0.05, †† p≤0.01, and ††† p≤0.001 (two-way ANOVA).

All the tested major pro-inflammatory cytokines were significantly elevated with ageing *per se* regarding their mRNA level (TNF 5-fold, p≤0.01; IL-1α ∼1.9-fold, p≤0.05; IL-1β ∼2.4-fold, p≤0.01; Figure 2). This up-regulation was reflected in the level of protein, though it was not as pronounced (TNF 1.1-fold, n.s.; IL-1α 1.1-fold, n.s.; Figure 2).

Following ischemia in adult mice brains, the transcript expression of all the tested pro-inflammatory cytokines was clearly elevated (TNF up to 61-fold, p≤0.001; IL-1α up to 13-fold, p≤0.01; IL-1β up to 37-fold, p≤0.001; Figure 2). This massive inflammatory response in adult ischemic brains was markedly attenuated in aged brains (by 41% for TNF; by 68% for IL-1α; by 79% for IL-1β).

The up-regulation of TNF mRNA and protein peaked at 12 h after reperfusion in adult mice (mRNA 56-fold, protein 2.3-fold; p≤0.001). The TNF protein level in aged mice brains following stroke showed an elevated expression but was not significantly affected. The extent of the post-ischemic response was approximately the same for IL-1α mRNA and protein, but a delayed peak expression of IL-1α protein was observed (mRNA peak at 6 h, protein peak at 12 h after reperfusion). The protein level of IL-1β could not be reliably quantified (data not shown).

### Cytokine IL-6

**Figure 3:**
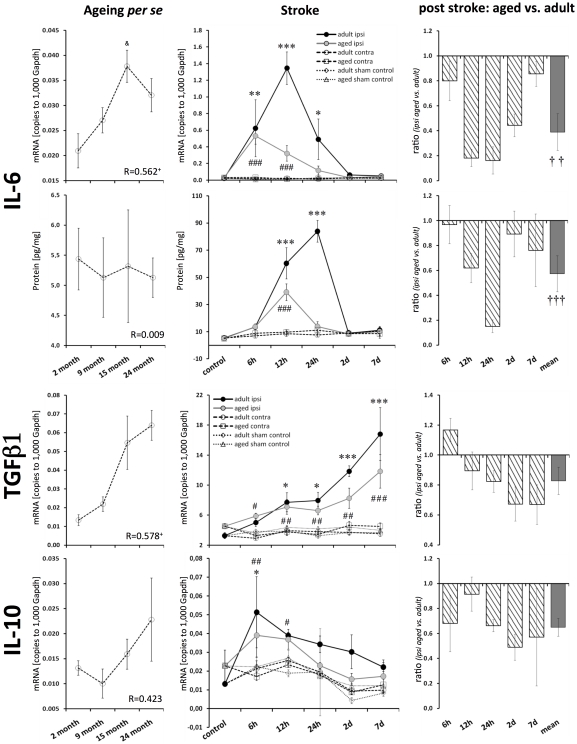
Age- and stroke-dependent expression of IL-6, TGFβ1 and IL-10. IL-6 transcript expression significantly correlated with age; however, this correlation was not reflected in the expression of its protein. The post-ischemic IL-6 response was attenuated in aged brains (mRNA and protein). TGFβ1 mRNA significantly correlated with age. IL-10 mRNA tended to be elevated in aged brains. TGFβ1 transcript expression in the ischemic tissue increased continuously with reperfusion time at both ages. The up-regulation of TGFβ1 and IL-10 following ischemia tended to be attenuated in aged brains. Expression values are shown as mean ± SEM and ratios as geometric mean ± SEM. For details of the statistical analysis, see Figure 2's legend or the Materials and Methods section.

The increased IL-6 mRNA level significantly correlated with age (Pearson, p≤0.05), though this was not reflected in its protein level (Figure 3). The post-ischemic IL-6 expression of adult mice was up-regulated by up to 69-fold for its mRNA level (peak at 12 h after reperfusion, p≤0.001) and by up to 11-fold for its protein level (peak at 24 h after reperfusion, p≤0.001; Figure 3). The ischemic response in aged brains was attenuated by 82% at the transcript level (at 12 h) and by 85% at the protein level (at 24 h). In adult mice, the mRNA expression of IL-6 increased from 6 h to 12 h when it reached its peak expression. In aged mice, IL-6 mRNA expression peaked at 6 h and then displayed a rapid decay.

### Anti-inflammatory cytokines IL-10 and TGFβ1

TGFβ1 transcript levels correlated significantly with age (Pearson, p≤0.05; Figure 3). In contrast to all the other tested cytokines, the post-ischemic TGFβ1, expression increased continuously with time (up to 7 d) in adult and aged brains (4.5-fold and 2.5-fold, respectively; p≤0.001). However, the up-regulation of TGFβ1 was attenuated in aged mice by 33% (7 d after reperfusion; Figure 3).

The expression of IL-10 mRNA tended to be elevated during ageing *per se*, but it was not significantly up-regulated (1.5-fold, n.s.; Figure 3). Following stroke, the IL-10 transcript expression peaked at 6 h after reperfusion for both ages of mice (2.1-fold, adult p≤0.05, aged p≤0.01). The post-ischemic IL-10 mRNA response was not found to be significantly altered in aged brains (Figure 3).

The protein expression of IL-10 and TGFβ1 could not be validated. The IL-10 protein levels in the test samples have been found below the sensitivity of the BD Bioscience Flex Set. In the case of TGFβ1, an appropriate antibody was not provided by the BD Bioscience Flex Set.

### Chemokines Mip-1α, MCP-1, and RANTES

**Figure 4:**
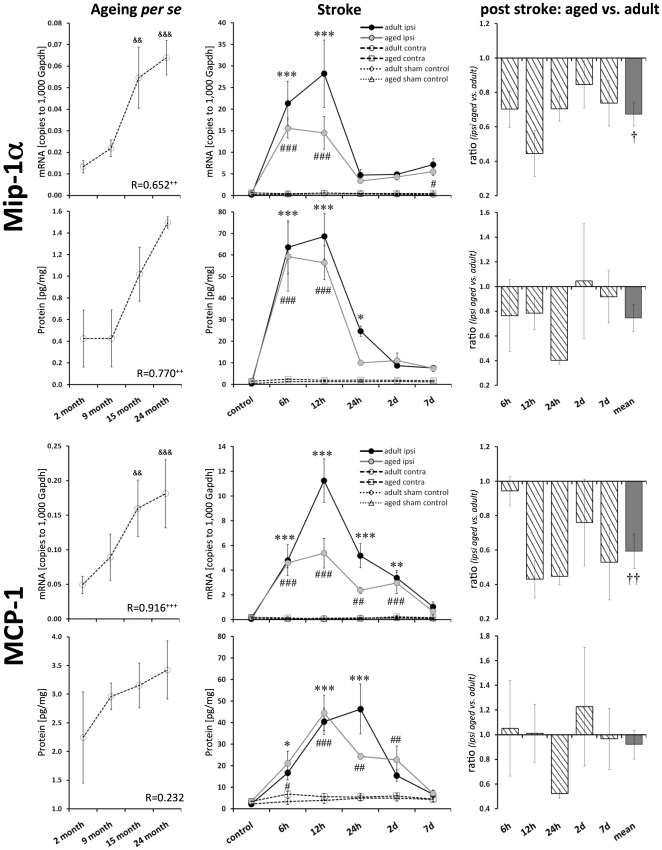
Age- and stroke-dependent expression of Mip-1α and MCP-1. Mip-1α and MCP-1 displayed significantly elevated expression levels with age. Mip-1α and MCP-1 transcript and protein expression increased significantly following stroke. This up-regulation was attenuated in aged brains. Expression values are shown as mean ± SEM and ratios as geometric mean ± SEM. For details of the statistical analysis, see Figure 2's legend or the Materials and Methods section.

**Figure 5:**
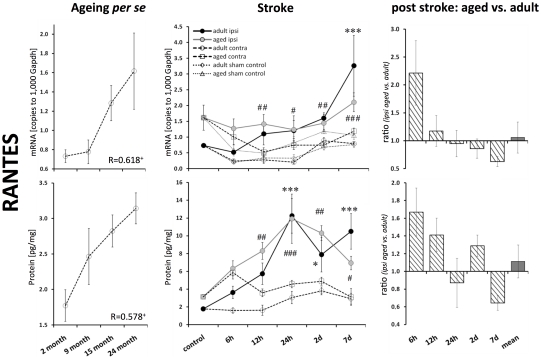
Age- and stroke-dependent expression of RANTES. The expression of RANTES significantly correlated with age. After ischemia, the level of RANTES mRNA continuously increased up to 7 d in adult brains, whereas no up-regulation was observed in aged brains. Protein levels of RANTES were up-regulated in adult and aged brains. Expression values are shown as mean ± SEM and ratios as geometric mean ± SEM. For details of the statistical analysis, see Figure 2's legend or the Materials and Methods section.

The transcript levels of all the tested chemokines increased with ageing *per se*. The strongest increase was observed for Mip-1α mRNA (3.5-fold, p≤0.001; Figure 4) and MCP-1 mRNA (3.6-fold, p≤0.001; Figure 4). The protein levels of Mip-1α and RANTES correlated significantly with age (Pearson, p≤0.01 and p≤0.05, respectively; Figures 4 and 5). The protein level of MCP-1 tended to be increased; however, it failed to reach significance (Figure 4).

Mip-1α mRNA expression peaked 12 h after ischemia in adult mice (Figure 4). Aged mice displayed a significant attenuated response of Mip-1α transcript expression at 12 h after reperfusion (by 55%) and over all time points (by 33%). This post-ischemic diminished Mip-1α response was observed to be less pronounced at the protein level (by 25%, n.s.).

The transcript expression of MCP-1 peaked at 12 h after ischemia in adult mice brains (up to 102-fold, p≤0.001; Figure 4). However, this strong response was significantly attenuated in aged mice brains (up to 57%). The post-ischemic protein expression peaked at 12 h and 24 h in aged and adult mice brains, respectively (up to 9-fold, adults, p≤0.001). MCP-1 mRNA response seems also be attenuated, in aged mice brains (by 48%, 24 h after reperfusion).

The expression of RANTES mRNA increased continuously up to 7 d following ischemia in adult brains (3.5-fold, p≤0.001; Figure 5). Aged brains showed no obvious post-ischemic increase in RANTES transcript expression in the ipsilateral hemisphere; however, the contralateral hemisphere displayed a decrease (Figure 5). Therefore, the ratio of ipsilateral versus contralateral RANTES mRNA resulted in a significant ischemic effect (Figure 5). The level of RANTES protein peaked at 24 h after reperfusion. RANTES did not display a significant age-dependent post-ischemic response (Figure 5).

## Discussion

**Figure 6:**
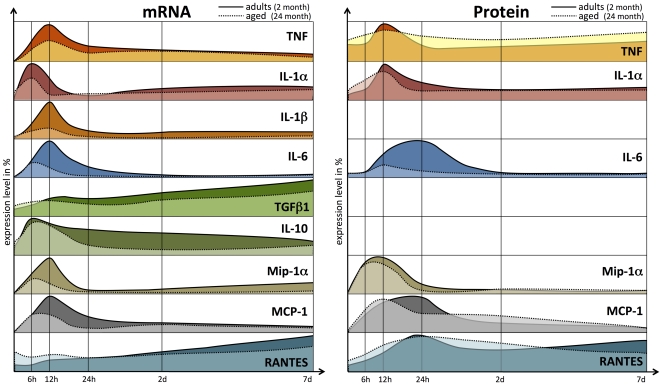
Schematic diagram summarizing the post-ischemic expression profiles of all the tested inflammatory mediators. The relative expression profiles (%) of all the tested inflammatory mediators in adult (continuous line, colored area) and aged brains (dotted line, light colored area) are shown (mRNA, left panel; protein, right panel). The post-ischemic response of TNF, IL-1α, IL-1β, IL-6, Mip-1α, MCP-1, and TGFβ1 was considerably attenuated with age, with IL-6 exhibiting the strongest age-dependent effect.

We found profound age-related alterations in the reaction to stroke. The response of pro-inflammatory cytokines and the level of chemokines were strongly diminished in the aged post-ischemic brain tissue (summarized in Figure 6). Anti-inflammatory cytokines (TGFβ1, and IL-10) revealed no significant age dependency after ischemia. In concordance, the mean infarct volume of aged mice brains was found to be smaller.

### Age dependence of infarct size

Previous studies reported contradictory results on the age dependence of stroke size. Most studies found no age dependence or rather smaller infarct sizes, in experimental [28], [29], [30] as well as in clinical [31], [32] observations. Larger stroke sizes in aged animals were reported after permanent MCAO at early times (6 h [33], 24 h [34], and 3 d [35]) while this was not observed at later times (7 d [35]), indicating that the dynamics of stroke development following permanent MCAO might be different in aged animals. Stroke size might also depend on the gender of aged animals and the hormone level. Female aged rodents had larger infarcts whereas ovariectomized rats had smaller infarcts [36], [37]. Altogether, our result of a tendency to smaller infarcts in aged mice following transient MCAO is in agreement with most of the previous experimental studies [28], [29], [30] and in line with clinical observations [31], [32].

### Elevated cerebral inflammation with age

The expression levels of inflammatory mediators in the brain are low due to the immune privilege of the central nervous system [38]. We therefore carefully established our qPCR settings to guarantee high sensitivity [27]. Protein expression was determined by flow cytometry with a bead-based immunoassay to minimize technical variations and to guarantee a high performance of each antibody in a multiplex scenario.

With the systematic approach used in this study, we showed that baseline expression levels of all the tested inflammatory mediators increased with age. Transcripts of the pro-inflammatory cytokines IL-1α, IL-1β, IL-6, and notably TNF as well as transcripts of the chemokines Mip-1α, MCP-1, and RANTES were up-regulated. Moreover, we found increased levels of the anti-inflammatory cytokines IL-10 and TGFβ1, with the transcript of TGFβ1 exhibiting the more prominent increase with age. These data are in agreement with the findings of previous studies, which reported similar tendencies for selected inflammatory mediators using different techniques [7], [9], [17], [18], [39]. With the exception of IL-6 and MCP-1, the protein expression of the inflammatory mediators increased with age, similar to their mRNA profiles. Although several methodological precautions were applied, the physiological protein levels were at (IL-6 and MCP-1) or below (IL-1β and IL-10) the limit of quantification. As mentioned above, an appropriate TGFβ1 antibody was not available.

### Attenuated inflammatory response after ischemic injury in aged brains

The post-ischemic response of the pro-inflammatory cytokines TNF, IL-1β, and particularly IL-6 as well as of the chemokines Mip-1α and MCP-1 was significantly attenuated in aged brains. The anti-inflammatory capacity (TGFβ1, and IL-10) revealed no significant age related differences after ischemia.

In line with our findings, an attenuated inflammatory response in aged brains was also observed following NMDA-induced brain injury (striatal TNF and cortical IL-1β [7]) or after whole brain irradiation (hippocampal TNF, IL-1β, IL-6, and MCP-1 [40]). In contrast, elevated pro-inflammatory reactions were observed in aged brains after mechanical injuries [9], [41], intracerebral hemorrhage [42], or ischemia [43]. Therefore, post-injury inflammatory mechanisms do not follow a general pattern, but appear to depend on the type and extent of stimulation.

### Potential reasons for the age-related attenuated post-ischemic inflammatory response

Elevated pro-inflammatory cytokine levels in aged brains may mediate a kind of preconditioning similar to the situation where administration of low levels of TNF and IL-1 leads to ischemic tolerance [44], [45]. In such a scenario, greater levels of stimulation would be required to induce a comparable inflammatory response. However, if and to what extent the attenuated post-ischemic immune response is due to elevated baseline levels of pro-inflammatory cytokines in aged brains cannot be determined from our results.

IL-6 exhibited the strongest decrease in its expression profile following stroke in aged mice brains. IL-6 is a pleiotropic cytokine that coordinates inflammatory processes between the periphery and the central nervous system, and can be released by various cell types in response to injuries. Moreover, IL-6 influences the expression and function of several other inflammatory mediators. Therefore, IL-6 may be a key mediator of the reduced inflammatory reaction following stroke in aged mice.

Similar to IL-6, all the other tested inflammatory mediators interact with each other. TNFα, for instance, influences several other inflammatory mediators, e.g. IL-6 and IL-1β [46], [47]. Also, IL-1β can stimulate cells to express MCP-1 [48]. Conversely, MCP-1-deficient mice express less IL-1β after permanent MCAO, indicating a signaling in both directions [49]. Therefore, a down-regulation of some of the key players of the post-stroke immune response may subsequently lead to a general reduction of the involved inflammatory mediators.

### Potential functional consequences of an attenuated inflammatory response

Pro-inflammatory cytokines are generally considered to mediate detrimental effects following ischemia. Administration of TNF or IL-1 exacerbates damage [19], [50], and inhibition of TNF or IL-1 as well as IL-1 deficiency lead to reduced ischemic injuries [19].

IL-6 as a pleiotropic mediator can potentially exert detrimental (early phase) or beneficial effects (late phase) following ischemia. Post-ischemic injection of recombinant IL-6 mediates neuroprotection and reduces the injury, whereas administration of anti-mouse IL-6 receptor monoclonal antibody or the use of IL-6-knockout mice increases infarct size [51].

Anti-inflammatory cytokines such as TGFβ1 and IL-10 have a beneficial function after ischemia. IL-10 deficiency exacerbates damage, whereas its over-expression reduces infarct volumes after ischemic brain injury [52]. Both of these anti-inflammatory cytokines are able to block NF-kappa B activation and thereby the expression of transcripts involved in immune and inflammatory responses [53], [54].

CC chemokines or β-chemokines recruit immune competent cells to injured tissue [55]. Studies which inhibited MCP-1 in the rat brain or used MCP-1-deficient mice reported smaller ischemic lesions, whereas over-expression exacerbated damage [19]. Inhibition of MCP-1 and Mip-1α signaling leads to reduced infarct volumes following MCAO [56]. In concordance, the administration of Mip-1α has been found to increase the infarct volume [56]. RANTES-deficient mice exhibited decreased leukocyte adhesion, diminished blood-brain barrier permeability, and less tissue infarction [57].

The present study has revealed an attenuated response of inflammatory mediators in aged brains following stroke. Moreover, we observed smaller infarcts in aged brains. Most studies in rodents as well as in humans have reported that cerebral infarcts in aged organisms are not different from those in adults [8]. The smaller infarcts in aged brains observed here could be a consequence of the diminished pro-inflammatory response. This hypothesis is in line with the literature which reports a detrimental effect of pro-inflammatory cytokines and chemokines on the extent of injuries (as outlined above). The attenuated expression of IL-6 in aged post-ischemic brains may be harmful in the late ischemic phase.

### Conclusions and future perspectives

From a theoretical point of view, attenuated post-lesional levels of pro-inflammatory cytokines and chemokines may be considered as a neuroprotective mechanism in aged brains. The smaller infarcts in aged brains could potentially be a consequence of this neuroprotection. However, reasons for the attenuated age-related stroke response cannot yet be specified. Increased baseline inflammation in aged brains may influence post-ischemic inflammatory processes. Modulation of the age-related neuro-inflammation (e.g. by pharmacological intervention or physical activity) with subsequent stroke induction would provide an opportunity to clarify this issue. Furthermore, investigations involving aged transgenic mice with altered inflammatory brain responses are necessary to unravel the functional relevance of our findings.

## Supporting Information

Supplement S1 (DOC)
