# Supplementary material for: Attenuated Inflammatory Response in Aged Mice Brains following Stroke
Source: PLoS One. 2011 Oct 18;6(10):e26288. doi: 10.1371/journal.pone.0026288 (PMC3196544; doi:10.1371/journal.pone.0026288)

**Supplemental information**

| **Age [months]** | | **2** | | | | | | **9** | **15** | **24** | | | | | |
| --- | --- | --- | --- | --- | --- | --- | --- | --- | --- | --- | --- | --- | --- | --- | --- |
| mRNA | Native | 4 | | | | | | 4 | 4 | 4 | | | | | |
| Protein |  | 4 | | | | | | 4 | 4 | 4 | | | | | |
| **Reperfusion time** | | **2h** | **6h** | **12h** | **24h** | **2d** | **7d** | **-** | **-** | **2h** | **6h** | **12h** | **24h** | **2d** | **7d** |
| Infarct volume | MCAO | 10 | - | - | - | 11 | 13 | - | - | 12 | - | - | - | 15 | 18 |
| mRNA | sham | - | 4 | 4 | 4 | 4 | 4 | - | - | - | 4 | 4 | 4 | 4 | 4 |
|  | MCAO | - | 4 | 4 | 4 | 4 | 4 | - | - | - | 4 | 4 | 4 | 4 | 4 |
| Protein | MCAO | - | 4 | 4 | 4 | 4 | 4 | - | - | - | 4 | 4 | 4 | 4 | 4 |

**Sample size calculation and exclusion criteria**

The number of animals needed to study the age dependence of the inflammatory response after stroke was calculated based on (a) mortality during ageing *per se*, (b) mortality after stroke and (c) variability of lesion size.

1. During ageing *per se*, 100% of mice achieved an age of 2 months, 92% of mice 9 months, 88% of mice 15 months and 79% of mice achieved an age of 24 months.
2. After stroke, 7% of all adult mice (2 month) and 39% of aged mice (24 month) died.
3. About 20% of mice displayed no signs of ischemia (Map2 staining). For expression studies (inflammation), about 50% were excluded because the infarct size was to small or to large. All animals with ischemia were included to measure the infarct volume.

In sum, three times as many mice (about 700) were needed than used for evaluation (231).

**Number of included mice**

**Randomization and allocation strategy**

Breeding and care of all mice, allocation of mice to the corresponding groups and induction of MCAO as well as sham surgery were carried out by separate investigators. Thus, all animals were randomly assigned to the ageing and treatment groups by an independent investigator without knowledge about the physical condition, behavior or any other aspects of appearance to avoid unwanted inclusion criteria’s. No animal was reallocated to another group after the study was started (e.g. we did not reallocated to early sacrifice group due to poor health).

**Blinded Assessment of Outcome**

Due to obviously phenotypic changes with age, the investigator responsible for surgery could not be blinded. Nevertheless, the person who did the surgery remained unclear concerning the subsequent analyses. Another investigator analyzed the infarct volumes, also without knowledge about the expected effects. The gene expression analyses were carried out by a liquid handling system and for protein expression analysis all samples were block randomized.

**Study design**

The study focused on inflammatory processes (1) during ageing *per se* and (2) following stroke in mice at different ages.

1. All animals were randomly assigned to the ageing groups: 2 months adults, 9 months middle aged, 15 and 24 months aged.


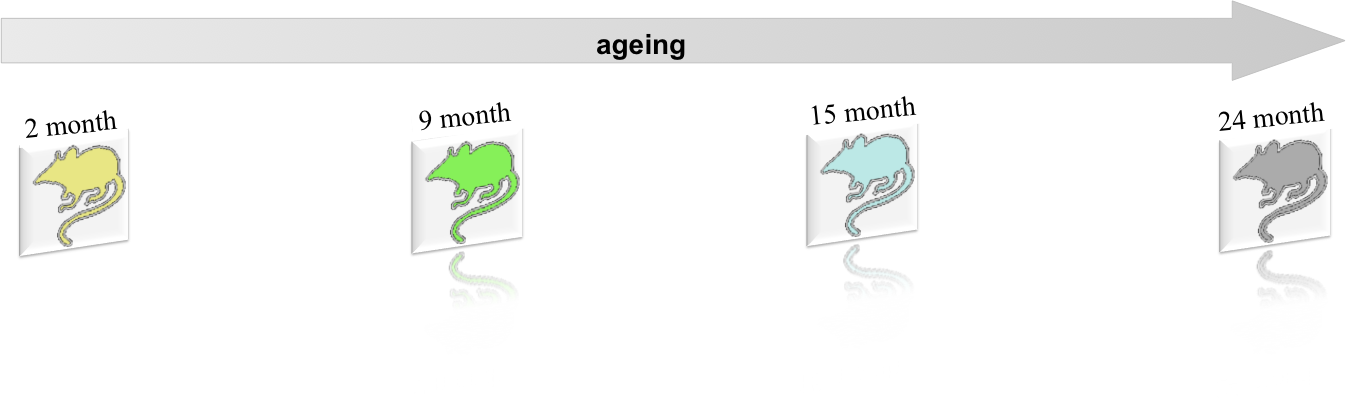


1. All animals were randomly assigned to the ageing groups: 2 months adults and 24 months old aged, the reperfusion times 6h, 12h, 24h, 2 and 7days as well as to the treatment groups MCAO and sham.


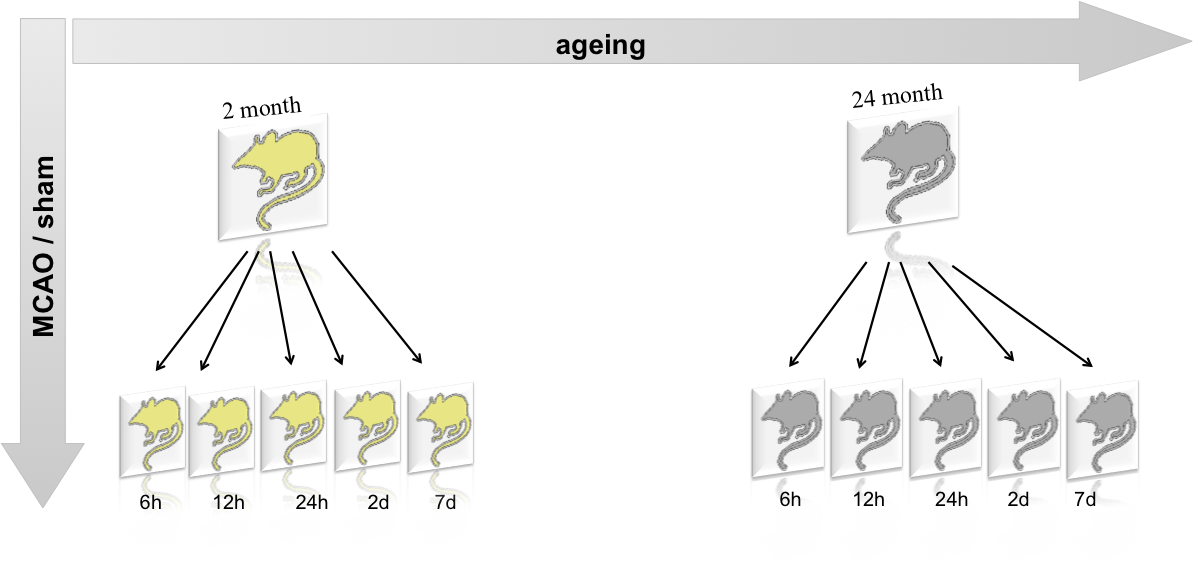

Supplement: Supplement S1 — (DOC) [file pone.0026288.s001.doc]
